# Dynamic cerebral autoregulation is intact in chronic kidney disease

**DOI:** 10.14814/phy2.15495

**Published:** 2022-11-02

**Authors:** Justin D. Sprick, Toure Jones, Jinhee Jeong, Dana DaCosta, Jeanie Park

**Affiliations:** ^1^ Division of Renal Medicine, Department of Medicine Emory University School of Medicine Atlanta Georgia USA; ^2^ Department of Veterans Affairs Health Care System Decatur Georgia USA; ^3^ Department of Kinesiology, Health Promotion and Recreation University of North Texas Denton Texas USA

**Keywords:** cerebral blood flow, cerebrovascular disease, renal disease, transfer function analysis

## Abstract

Chronic Kidney Disease (CKD) patients experience an elevated risk for cerebrovascular disease. One factor that may contribute to this heightened risk is an impairment in dynamic cerebral autoregulation, the mechanism by which cerebral vessels modulate cerebral blood flow during fluctuations in arterial pressure. We hypothesized that dynamic cerebral autoregulation would be impaired in CKD. To test this hypothesis, we compared dynamic cerebral autoregulation between CKD patients stages III‐IV and matched controls (CON) without CKD. Fifteen patients with CKD and 20 CON participants performed 2, 5‐minute bouts of repeated sit‐to‐stand maneuvers at 0.05 Hz and 0.10 Hz while mean arterial pressure (MAP, via finger photoplethysmography) and middle cerebral artery blood velocity (MCAv, via transcranial Doppler ultrasound) were measured continuously. Cerebral autoregulation was characterized by performing a transfer function analysis (TFA) on the MAP‐MCAv relationship to derive coherence, phase, gain, and normalized gain (nGain). We observed no group differences in any of the TFA metrics during the repeated sit‐to‐stand maneuvers. During the 0.05 Hz maneuver, Coherence: CKD = 0.83 ± 0.13, CON = 0.85 ± 0.12, Phase (radians): CKD = 1.39 ± 0.41, CON = 1.25 ± 0.30, Gain (cm/s/mmHg): CKD = 0.69 ± 0.20, CON = 0.71 ± 0.22, nGain (%/mmHg): CKD = 1.26 ± 0.35, CON = 1.20 ± 0.28, *p* ≥ 0.24. During the 0.10 Hz maneuver (*N* = 6 CKD and *N* = 12 CON), Coherence: CKD = 0.61 ± 0.10, CON = 0.67 ± 0.11, Phase (radians): CKD = 1.43 ± 0.26, CON = 1.30 ± 0.23, Gain (cm/s/mmHg): CKD = 0.75 ± 0.15, CON = 0.84 ± 0.26, nGain (%/mmHg): CKD = 1.50 ± 0.28, CON = 1.29 ± 0.24, *p* ≥ 0.12. Contrary to our hypothesis, dynamic cerebral autoregulation remains intact in CKD stages III‐IV. These findings suggest that other mechanisms likely contribute to the increased cerebrovascular disease burden experienced by this population. Future work should determine if other cerebrovascular regulatory mechanisms are impaired and related to cerebrovascular disease risk in CKD.

## Graphical abstract

Chronic Kidney Disease (CKD) patients experience an elevated risk for cerebrovascular disease, which may be mediated in part by an impairment in dynamic cerebral autoregulation. We compared dynamic cerebral autoregulation between CKD patients stages III‐IV vs matched controls (CON) without CKD and hypothesized that dynamic cerebral autoregulation would be impaired in CKD. Contrary to our hypothesis, dynamic cerebral autoregulation remains intact in CKD, which suggests that other mechanisms likely contribute to the increased cerebrovascular disease burden experienced by this population.

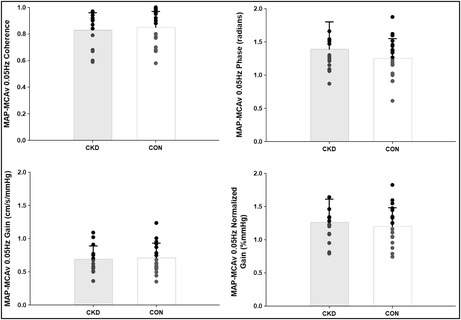

## 1 INTRODUCTION

Patients with chronic kidney disease (CKD) have a substantially elevated risk for cerebrovascular disease including stroke (Lee et al., 2010), transient ischemia attack (Koren‐Morag et al., 2006), and cerebral small vessel disease (Ikram et al., 2008). Moreover, when CKD patients do experience a stroke, they suffer from higher mortality rates (Tsagalis et al., 2009). One factor that may contribute to heightened cerebrovascular risk in CKD is an impairment in dynamic cerebral autoregulation, that is, the mechanism through which the cerebral vasculature stabilizes cerebral blood flow during fluctuations in arterial pressure (Claassen et al., 2021). Other disease states characterized by increased stroke risk (e.g., atrial fibrillation, malignant hypertension, diabetes) exhibit impaired cerebral autoregulation (Immink et al., 2004; Junejo et al., 2020; Vianna et al., 2015), and this is clinically relevant because impairments in cerebral autoregulation predict poor functional outcomes following stroke (Chi et al., 2018). If cerebral autoregulation is impaired in CKD, then this may comprise an important therapeutic target for the prevention of cerebrovascular disease in this population.

There is limited knowledge regarding how cerebrovascular regulatory mechanisms, including cerebral autoregulation, are affected by renal disease (Sprick et al., 2020). One animal study demonstrated impaired autoregulation in cerebral vessels excised from uremic rats (New et al., 2003), but there have been very few human studies to extend this finding. The few human studies that have been performed have focused almost exclusively on end‐stage renal disease (ESRD) patients undergoing hemodialysis, and have not included patients with mild to moderate impairments in kidney function and not on renal replacement therapy (Sprick et al., 2020). One recent investigation did report an association between impaired cerebral autoregulation and reduced estimated glomerular filtration rate (eGFR) in patients treated for ischemic stroke (Castro et al., 2018), suggesting that cerebral autoregulation may contribute to the heightened stroke risk in CKD. However, cerebral autoregulation is known to be impaired following stroke (Aries et al., 2010; Intharakham et al., 2019), and it remains unclear if impaired cerebral autoregulation contributed to stroke in these patients, or if stroke‐induced impairments in cerebral autoregulation are amplified in the setting of CKD. We aimed to determine if cerebral autoregulation is impaired in patients with CKD stages III‐IV without a history of cerebrovascular disease compared to a control (CON) group free from both renal and cerebrovascular disease. We hypothesized that cerebral autoregulation would be impaired in CKD patients stages III‐IV.

## 2 METHODS

### 2.1 Ethics statement

This study was approved by the Atlanta Veterans Affairs (VA) Health Care System Research and Development Committee and the Emory University Institutional Review Board. Written informed consent was obtained for all study participants and all study procedures conformed to the standards set forth by the Declaration of Helsinki. This study is registered at clinicaltrials.gov (NCT 02947750).

### 2.2 Participants

Sedentary individuals with CKD stages III‐IV (eGFR between 15–59 ml/min/1.73 m^2^), as defined by the CKD‐EPI equation (Levey et al., 2006) were recruited from Emory University clinics and the Atlanta VA Health Care System for participation in this study. CON participants without CKD were also recruited from these same locations for participation in this study. CON participants were matched for age, race, sex, and hypertension and diabetes status to allow us to better isolate the effects of CKD in the presence of these common comorbidities. Exclusion criteria for both groups included uncontrolled hypertension (blood pressure >160/90 mmHg), vascular disease (including prior stroke), clinical evidence of heart failure or heart disease determined by electrocardiogram (ECG) or echocardiogram, engagement in regular exercise (defined as greater than 20 min of physical activity at least twice per week over the last 6 months), drug or alcohol abuse within the past 12 months, diabetic neuropathy, severe anemia (hemoglobin <10 mg/dl), and pregnancy.

### 2.3 Instrumentation

Participants reported to the human physiology laboratory at Emory University School of Medicine after abstaining from food, alcohol, and caffeine for at least 12 hours, and exercise for a minimum of 24 h. Participants taking medications took all medications as prescribed. Resting blood pressure and heart rate were measured in triplicate via an automated device (Omron, Hem907XL) in the seated position after 5 minutes of quiet rest with the arm supported at the heart level in accordance with the American College of Cardiology/American Heart Association Guidelines (Whelton et al., 2018).

Following the resting measurement, participants were instrumented for continuous measurement of heart rate (HR) via a standard three lead ECG (AD Instruments), and beat to‐beat arterial pressure via finger photoplethysmography (Finometer, Finapress Medical Systems) applied to the dominant hand. This arm was placed in a sling at heart level for stability to ensure accurate detection of the blood pressure waveform throughout the repeated sit‐to‐stand maneuvers. Respiration rate and end tidal CO_2_ (etCO_2_) were measured on a breath‐by‐breath basis through an oral‐nasal cannula via capnography (Gemini Respiratory Gas Analyzer CWE14‐10000, CWE Inc). Middle cerebral artery blood velocity (MCAv) was measured via transcranial Doppler (TCD) ultrasound (Multi‐Dop, DWL). Specifically, 2 MHz probes were secured over the temporal window of the participants head via adjustable headgear (Diamon, DWL) while MCAv was acquired using a standardized approach as outlined in the literature (Willie et al., 2011). Efforts were made to ensure that MCAv recordings were obtained from the left side of the head in all participants; however, the side with the best signal quality was used for analysis.

### 2.4 Experimental protocol

**FIGURE 1:**
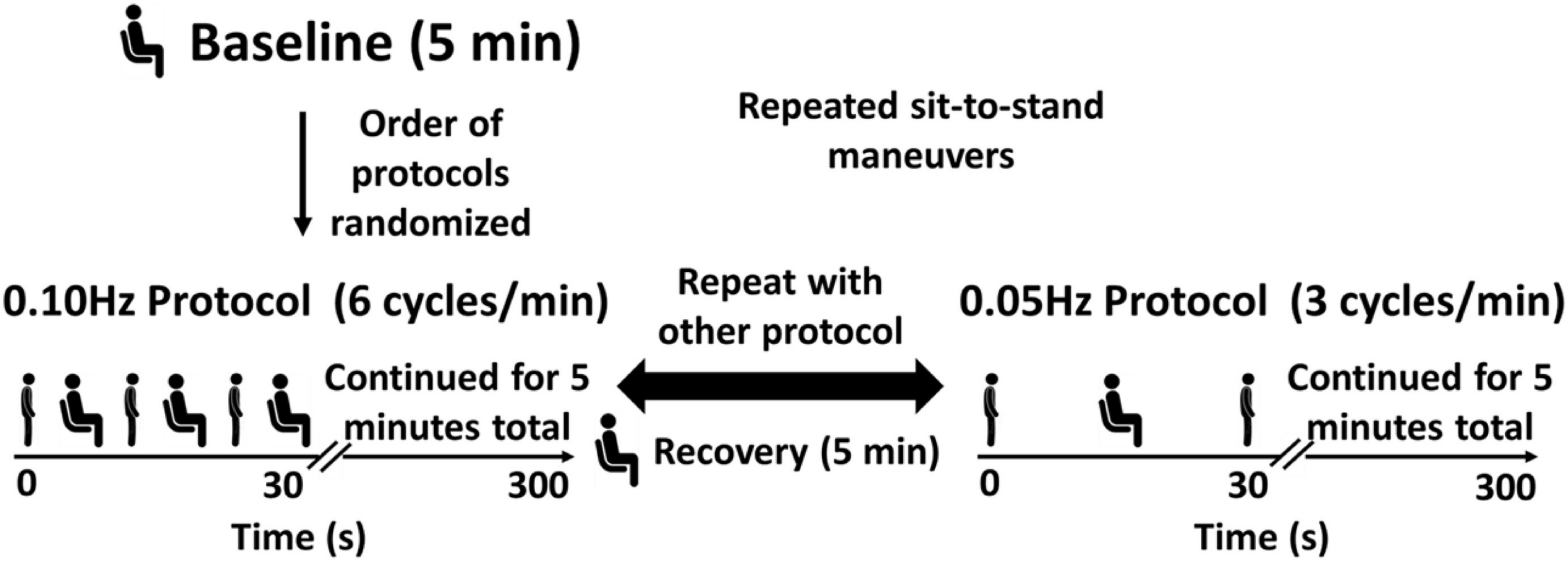
Experimental Timeline

Cerebral autoregulation was assessed during repeated bouts of sit‐to‐stand, performed at 0.05 Hz and 0.10 Hz (Smirl et al., 1985). This approach mimics the postural stress imposed during daily activities when cerebral autoregulation is challenged and has been used to measure cerebral autoregulation in related populations (de Heus et al., 2018; van Beek et al., 2010). Following instrumentation, a 5‐minute seated baseline was observed, after which participants performed the two, 5‐minute bouts of repeated sit‐to‐stand maneuvers at a 0.05 Hz (10‐s sit, 10‐s stand) and 0.10 Hz (5 s‐sit, 5‐s stand). The sequence of sit‐to‐stand maneuvers was randomized, and a 5‐minute seated recovery period was observed between bouts to allow all cardiovascular parameters to return to baseline (Figure 1). A metronome was used to pace participants at these specific frequencies during the maneuvers, and they were instructed to breathe at their normal rate. Verbal cues were also given by the investigators to ensure that participants maintained the correct cadence of the driven frequency when necessary (e.g., 10s‐sit, 10‐s stand for 0.05 Hz, and 5‐s sit, 5‐s stand for 0.10 Hz).

### 2.5 Data analysis

Five‐minute averages were analyzed for each of the three timepoints of interest (e.g., baseline, 0.05 Hz sit‐to‐stand, and 0.10 Hz sit‐to‐stand). All continuous waveform data (e.g., ECG, arterial pressure, MCAv, respiratory rate, etCO_2_) were collected at 1000 Hz (Labchart, AD Instruments) and analyzed offline via specialized software (Elucimed). The timing of each cardiac cycle was determined from the R‐wave of the ECG signal and beat‐to‐beat arterial pressures and MCAv were then detected. Mean arterial pressure (MAP) and mean MCAv were automatically calculated as the area under the arterial pressure and cerebral blood velocity waveform. Cerebral autoregulation was assessed in accordance with standardized guidelines set forth by the Cerebrovascular Research Network (CARnet) (Claassen et al., 2016). Briefly, 300 s of beat‐to‐beat MAP and MCAv signals were spline interpolated, re‐sampled at 4 Hz, and fast Fourier transformed for spectral analysis and subsequent transfer function analysis (TFA) using the Welch algorithm. The five‐minute recordings were subdivided into five successive 100 second window segments which overlapped by 50% each. Each window segment was linearly detrended and passed through a Hanning window for linear transfer function analysis. The cross‐spectrum between MAP and MCAv was determined and divided by the MAP auto‐spectrum. MAP‐MCAv coherence, phase (radians), absolute gain (cm/s/mmHg), and normalized gain (nGain, %/mmHg) were then determined at the point estimates of the driven frequencies during the repeated sit‐to‐stand maneuvers (0.05 Hz and 0.10 Hz). These frequencies are within the very low frequency (VLF, 0.02–0.07 Hz) and low frequency (0.07–0.20 Hz) ranges in which dynamic cerebral autoregulation is operant (Smirl et al., 1985). Transfer function phase and gain were only assessed when the corresponding coherence reached the a priori defined 95% statistical significance level as is consistent with previous literature using this same methodology and recommended by standardized guidelines (Claassen et al., 2016). Phase wrap‐around was not present at any of the point‐estimate values during the repeated sit‐to‐stand maneuvers.

### 2.6 Statistical analysis

Participant demographic data, medication use, resting hemodynamics and cerebrovascular parameters, and resting MAP and MCAv spectral power within the very low frequency (VLF, 0.02–0.07 Hz) and low frequency (0.07–0.20 Hz) ranges were compared between groups via unpaired, two‐tailed, T‐tests for continuous variables, or chi‐square analysis for categorical variables. During each of the two repeated sit‐to‐stand maneuvers, MAP and MCAv spectral power and MAP‐MCAv transfer function coherence, phase, gain, and nGain were compared at the point estimates of the driven frequencies (0.05 Hz and 0.10 Hz) via unpaired, two‐tailed *t*‐tests. Normality of all data was confirmed via the Kolmogorov–Smirnov test. All data are presented as mean ± SD.

## 3 RESULTS

### 3.1 Participants

Twenty‐four patients with CKD stages III‐V and 24 controls free from renal disease were recruited to participate in this study. We were unable to acquire useable ultrasound signals for the MCA in 13 participants (*N* = 9 CKD, *N* = 4 CON) so these participants did not complete the experimental protocol and were thus excluded from analyses. As such, we obtained data from *N* = 15 CKD (mean eGFR = 39 ± 9 ml/min/1.73 m^2^, range = 15–51 ml/min/1.73 m^2^) and *N* = 20 CON for all measurements unless otherwise indicated. Demographic data for both groups are reported in Table 1. The majority of participants in each group were Black males. There were no differences in age, sex, weight, blood pressure, or the presence of diabetes between groups. Hypertension was common in both groups and most participants were taking anti‐hypertensive medications; however, there were no differences in anti‐hypertensive medication use between groups (*p* ≥ 0.08).

**TABLE 1:** Participant demographic data and medication use

Characteristic	CKD	CON	*p*
*n*	15	20	
Age (y)	64 ± 10	59 ± 9	0.14
eGFR (ml/min/1.73 m^2^)	39 ± 9	76 ± 13	<0.0001
Systolic arterial pressure	123 ± 18	121 ± 12	0.68
Diastolic arterial pressure	72 ± 8	75 ± 12	0.46
Sex (M/F)	10/5	12/8	0.69
Race, *n* (%)			0.25
Black	12 (80%)	15 (100%)	
White	3 (20%)	0 (0%)	
Body weight (kg)	93 ± 14	88 ± 19	0.40
Body mass index (kg m^−2^)	31.5 ± 4.4	29.8 ± 6.1	0.40
Diabetes, *n* (%)	6 (40%)	5 (25%)	0.34
Hypertension, n (%)	13 (87%)	12 (60%)	0.08
Anti‐hypertensive medications, *n* (%)			
Calcium channel blockers, *n* (%)	9 (60%)	6 (30%)	0.08
ACE inhibitors/ARBs, *n* (%)	9 (60%)	7 (35%)	0.40
Diuretics, *n* (%)	5 (33%)	5 (25%)	0.59
β‐blockers, *n* (%)	7 (47%)	4 (20%)	0.09
α‐blockers, *n* (%)	3 (20%)	1 (5%)	0.17
Hydralazine, *n* (%)	1 (7%)	1 (5%)	0.83

Abbreviations: ACE, angiotensin‐converting enzyme; ARB, angiotensin receptor blocker; CKD, Chronic Kidney Disease; CON, Control; eGFR, Estimated Glomerular Filtration Rate.

### 3.2 Baseline hemodynamics and cerebrovascular parameters

Resting hemodynamic and cerebrovascular parameters are provided in Table 2. Blood pressure measurements are elevated in both groups compared to resting values likely due to methodological differences in how the measurement was performed (Oscillometric cuff vs finger photoplethysmography). EtCO_2_ and respiration rate data are missing for *N* = 1 CON participant due to technical difficulties with the gas analyzer that day, thus data from *N* = 15 CKD and *N* = 19 CON for resting etCO_2_ and respiration rate data were obtained. There were no differences in resting heart rate, arterial pressure, MCAv or etCO_2_ between groups (*p* ≥ 0.15).

**TABLE 2:** Resting hemodynamic and cerebrovascular parameters during seated baseline prior to repeated sit‐to‐stand maneuvers

Characteristic	CKD	CON	*p*
*n*	15	20	
Heart rate (bpm)	75 ± 12	76 ± 11	0.79
Systolic blood pressure (mmHg)	144 ± 21	140 ± 14	0.43
Diastolic blood pressure (mmHg)	78 ± 14	78 ± 9	0.91
Mean arterial pressure (mmHg)	102 ± 15	102 ± 10	0.93
Middle cerebral artery mean blood velocity (cm/s)	57 ± 9	59 ± 15	0.55
Cerebrovascular resistance index (mmHg/cm/s)	1.9 ± 0.4	1.8 ± 0.5	0.67
Respiration rate (bpm)	15 ± 4	15 ± 3	0.75
End‐tidal partial pressure of carbon dioxide (mmHg)	35 ± 3	37 ± 3	0.15

Abbreviations: CKD, Chronic Kidney Disease; CON, Control.

### 3.3 Forced oscillations in mean arterial pressure and middle cerebral artery blood velocity

**FIGURE 2:**
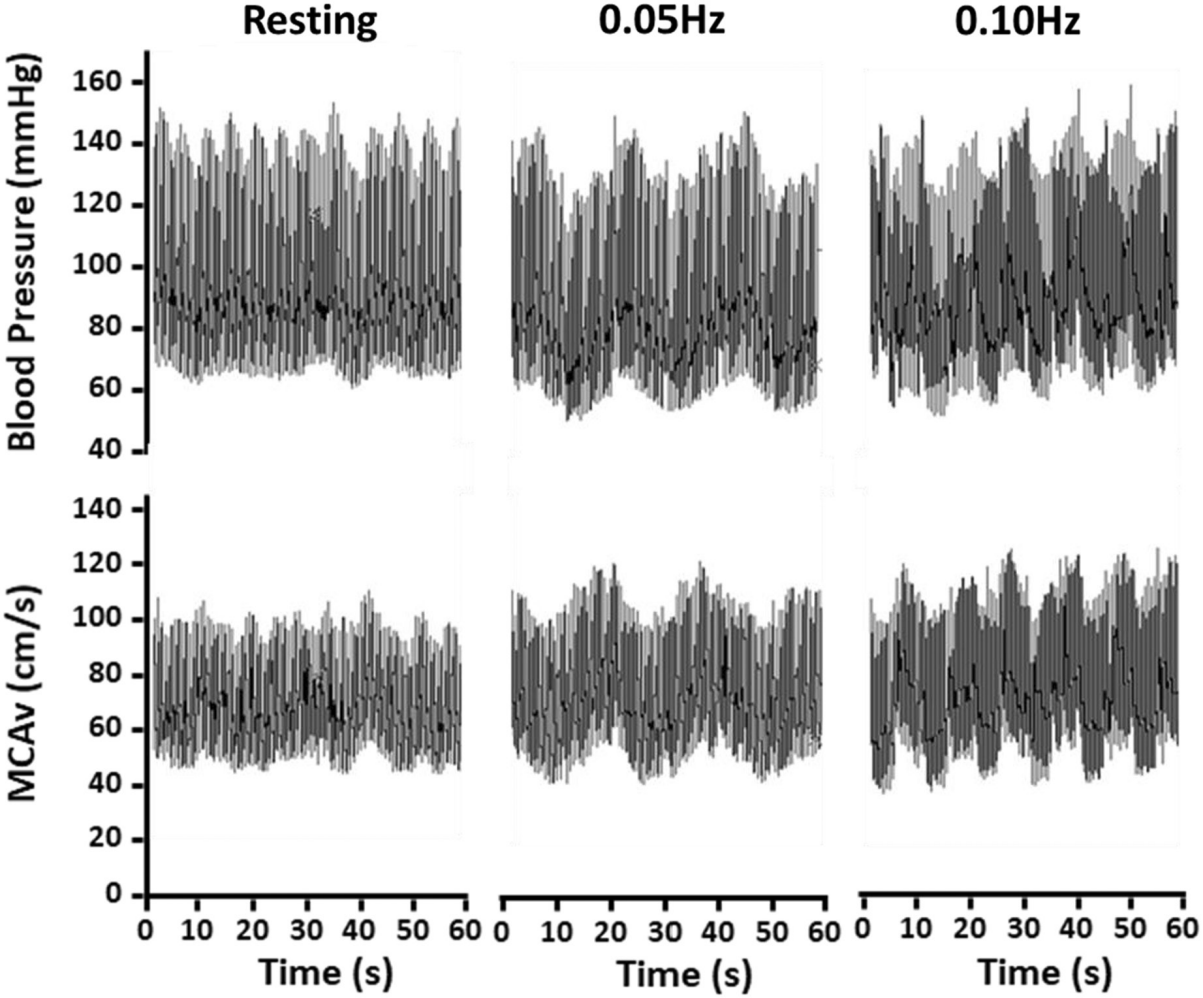
Representative blood pressure and middle cerebral artery blood velocity (MCAv) tracing during repeated bouts of sit to stand in control participant

The repeated sit‐to‐stand maneuvers induced robust oscillations in blood pressure and MCAv at their respective frequencies. Figure 2 shows representative recordings for blood pressure and MCAv collected from a CON participant at rest and during each of the repeated sit‐to‐stand maneuvers. As expected, MAP and MCAv spectral power increased at the driven frequencies during each of the maneuvers with no differences between groups (Table 3, *p* ≥ 0.52). EtCO_2_ remained stable throughout both maneuvers with no differences between groups (0.05 Hz: CKD = 35 ± 5 mmHg, CON = 38 ± 3 mmHg, *p* = 0.06; 0.10 Hz: CKD = 36 ± 5 mmHg, CON = 38 ± 4 mmHg, *p* = 0.11).

**TABLE 3:** Arterial pressure and middle cerebral artery blood velocity power spectral densities at rest and during repeated bouts of sit‐to‐stand maneuvers

Characteristic	CKD	CON	*p*
Rest			
*n*	15	20	
Very low frequency range (0.02–0.07 Hz)			
Mean arterial pressure power (mmHg^2^)	20.3 ± 18.9	22.1 ± 15.3	0.82
Middle cerebral artery blood velocity power (cm/s^2^)	7.2 ± 4.6	10.3 ± 7.1	0.16
Low frequency range (0.07–0.20 Hz)			
Mean arterial pressure power (mmHg^2^)	13.0 ± 12.5	7.1 ± 4.4	0.07
Middle cerebral artery blood velocity power (cm/s^2^)	4.8 ± 4.5	5.1 ± 4.9	0.90
0.05 Hz Sit‐to‐Stand			
*n*	15	20	
Mean arterial pressure power (mmHg^2^)	97.0 ± 87.7	87.1 ± 65.7	0.72
Middle cerebral artery blood velocity power (cm/s^2^)	42.1 ± 26.2	45.7 ± 35.2	0.75
0.10 Hz Sit‐to‐Stand			
*n*	6	12	
Mean arterial pressure power (mmHg^2^)	59.5 ± 37.5	68.5 ± 64.1	0.78
Middle cerebral artery blood velocity power (cm/s^2^)	39.2 ± 30.7	54.5 ± 51.8	0.52

Abbreviations: CKD, Chronic Kidney Disease; CON, Control.

**FIGURE 3:**
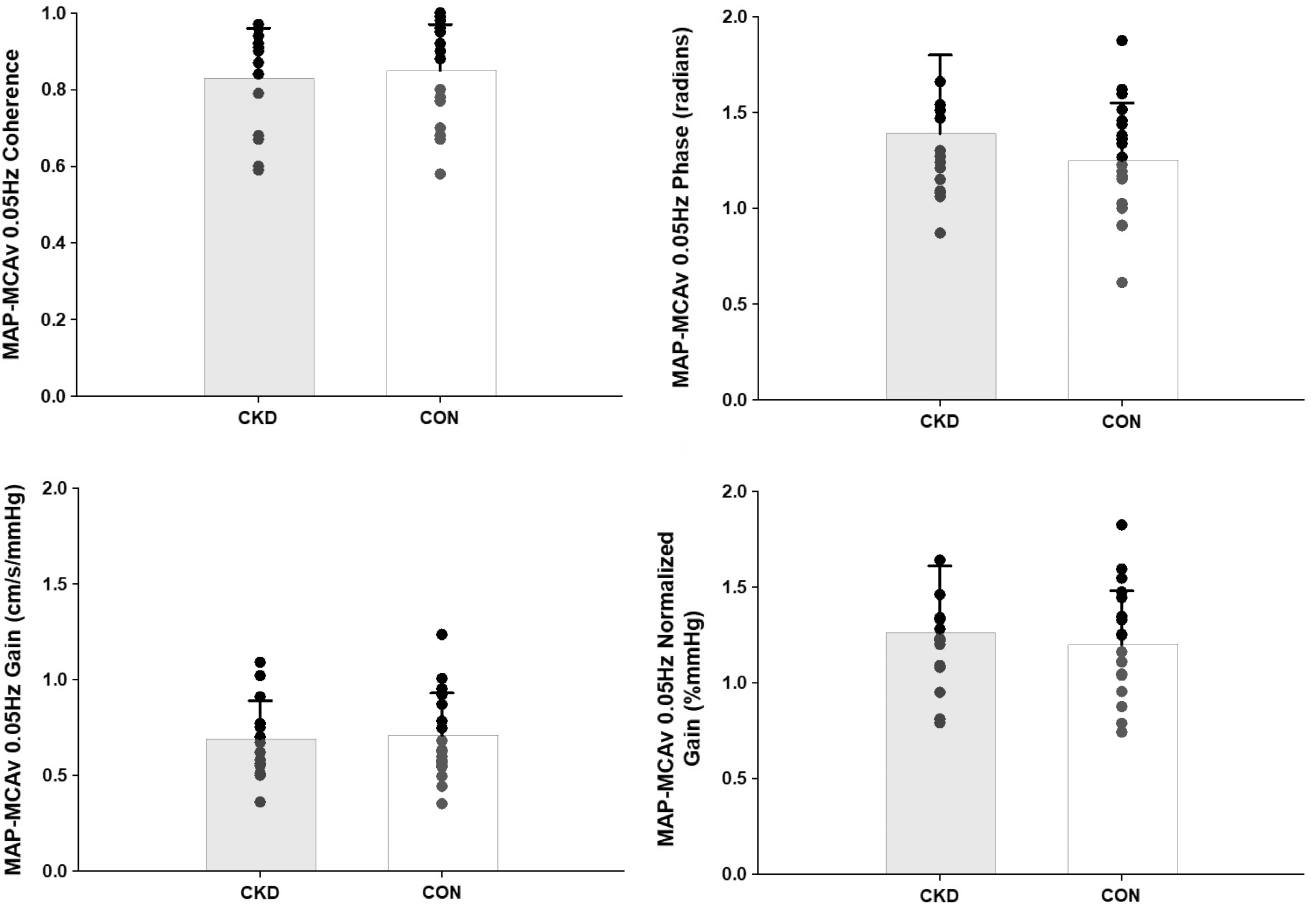
Transfer function analysis of forced oscillations in mean arterial pressure‐middle cerebral artery blood velocity (MAP‐MCAv) during repeated bouts of sit‐to‐stand at 0.05 Hz. *N* = 15 CKD (10 M/5F) and *N* = 20 CON (12 M/8F). MAP‐MCAv coherence, phase, gain, and normalized gain were compared between groups via two‐tailed, unpaired *t*‐tests. *p* ≥ 0.24 for all comparisons

**FIGURE 4:**
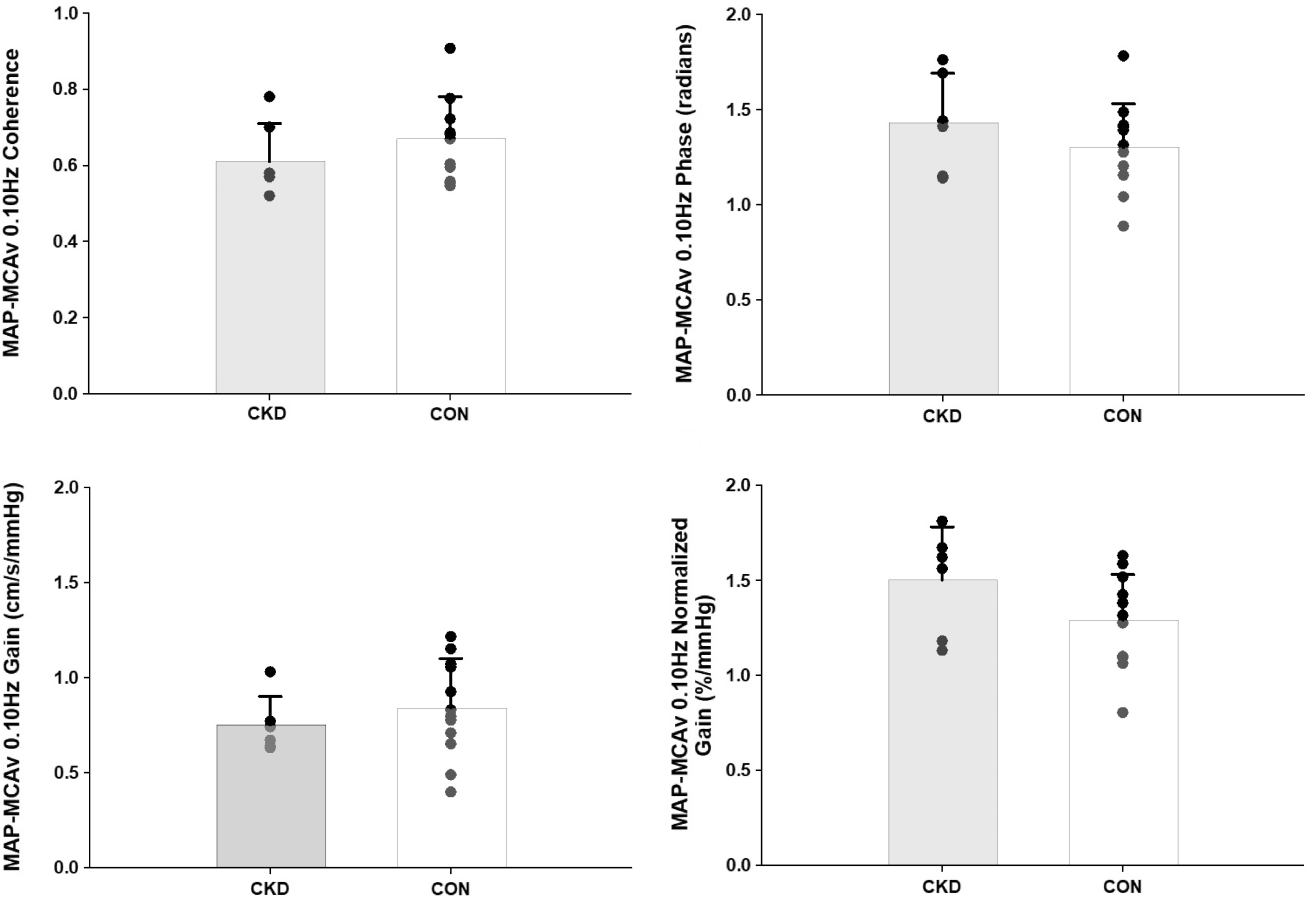
Transfer function analysis of forced oscillations in mean arterial pressure‐middle cerebral artery blood velocity (MAP‐MCAv) during repeated bouts of sit‐to‐stand at 0.10 Hz. *N* = 6 CKD (3 M/3F) and *N* = 12 CON (6 M/6F). MAP‐MCAv coherence, phase, gain, and normalized gain were compared between groups via two‐tailed, unpaired *t*‐tests. *p* ≥ 0.12 for all comparisons

TFA derived metrics for the MAP‐MCAv relationship are shown in Figures 3 and 4. There were no differences in coherence (CKD = 0.83 ± 0.13, CON = 0.85 ± 0.12, *p* = 0.67), phase (CKD = 1.39 ± 0.41, CON = 1.25 ± 0.30, *p* = 0.24), gain (CKD = 0.69 ± 0.20, CON = 0.71 ± 0.22, *p* = 0.81), or nGain (CKD = 1.26 ± 0.35, CON = 1.20 ± 0.28, *p* = 0.56) between groups during the 0.05 Hz maneuver (Figure 3). Not all participants were able to complete the 0.10 Hz maneuver due to musculoskeletal limitations that precluded their ability to complete this more strenuous, fast‐faced bout of repeated sit‐to‐stand (*N* = 5 CKD, *N* = 2 CON). Of the participants that did complete the 0.10 Hz maneuver, coherence remained <0.5 for *N* = 4 CKD and *N* = 6 CON, precluding our ability to analyze phase and gain metrics, so these participants were also excluded from the final analysis. As such, *N* = 6 CKD and *N* = 12 CON during 0.10 Hz repeated sit‐to‐stand. There were no group differences in MAP‐MCAv coherence (CKD = 0.61 ± 0.10, CON = 0.67 ± 0.11, *p* = 0.31), phase (CKD = 1.43 ± 0.26, CON = 1.30 ± 0.23, *p* = 0.30), gain (CKD = 0.75 ± 0.15, CON = 0.84 ± 0.26, *p* = 0.43), or nGain (CKD = 1.50 ± 0.28, CON = 1.29 ± 0.24, *p* = 0.12) between groups during the 0.10 Hz maneuver (Figure 4).

## 4 DISCUSSION

In this investigation, we compared dynamic cerebral autoregulation between CKD patients without a prior history of cerebrovascular disease to controls free from both renal and cerebrovascular disease. Contrary to our hypothesis, we observed no impairment in dynamic cerebral autoregulation during repeated bouts of sit‐to‐stand in CKD. These findings have important implications for stroke prevention in CKD and suggest that other mechanisms beyond cerebral autoregulation contribute to the increased cerebrovascular disease burden experienced in this population.

To the best of our knowledge, this is the first study to assess dynamic cerebral autoregulation in CKD without a prior history of stroke. Our findings contrast with a prior report by Castro et al., which observed impaired dynamic cerebral autoregulation in CKD patients treated for ischemic stroke (Castro et al., 2018). These disparate results may be due to methodological differences between studies and the presence of preexisting cerebrovascular disease. Specifically, Castro et al. relied on spontaneous oscillations in arterial pressure and MCAv to measure cerebral autoregulation while we used repeated bouts of sit to stand to drive these oscillations at the specific frequencies of interest (i.e. 0.05 Hz and 0.10 Hz). Further, Castro et al. studied CKD patients within 6 hours of stroke symptom onset (Castro et al., 2018) while we excluded patients with a history of cerebrovascular disease. Relating our findings to those reported by Castro et al., it appears that cerebral autoregulation is not impaired in CKD patients without a prior history of stroke, but stroke‐induced impairments in autoregulation may be amplified in the setting of CKD. This finding is surprising, given that other chronic disease states exhibit impairments in cerebral autoregulation (Immink et al., 2004; Junejo et al., 2020; Vianna et al., 2015). One potential explanation could be that the cerebral vasculature has adapted in CKD to cope with the increased blood pressure variability that CKD patients experience (Sarafidis et al., 2018). Studies performed in ESRD have primarily focused on the effects of hemodialysis on cerebral autoregulation and generally report that some degree of impairment is present (Sprick et al., 2020). Thus, it is possible that these impairments do not manifest until almost all residual kidney function has deteriorated or that the hemodialysis procedure itself alters the cerebral pressure‐flow relationship. Other potential cerebrovascular mechanisms that may link renal insufficiency to cerebrovascular disease risk include impairments in cerebrovascular CO_2_ reactivity and neurovascular coupling. Future studies should explore if these mechanisms are altered and related to future cerebrovascular disease risk in CKD.

There are a number of methodological considerations that should be mentioned as they relate to interpretation of our findings. First, we compared cerebral autoregulation between CKD patients and a control group matched for hypertension and diabetes and did not include a healthy control group for comparison. We contend that this approach is most appropriate since CKD rarely presents in isolation and hypertension and diabetes are the two leading causes of CKD. Further, inclusion of a completely healthy control group would result in differences in medication use between groups that could confound findings given the known effects of certain anti‐hypertensive medications on cerebral autoregulation. Specifically, calcium channel blockers, a first line anti‐hypertensive medication, interfere with autoregulation through an impairment in myogenic control (Castro et al., 2018; Tan et al., 2013). Second, our patient population consisted of mostly males, so we were unable to explore potential sex differences. Prior work has suggested that females exhibit enhanced cerebral autoregulation relative to males (Deegan et al., 2009; Favre & Serrador, 2019) so future work should determine if these sex differences are also present in the setting of CKD. Additionally, due to the age (61 ± 9 years) and sedentary nature of our participants, not all participants were able to complete the 0.10 Hz sit‐to‐stand maneuver due to the strenuous nature of this fast‐paced maneuver. CKD participants were recruited as part of a larger ongoing clinical trial testing the effects of exercise training on neurovascular regulation in CKD. As regular exercise training was an exclusion criterion for that larger trial, participants included in this study were also sedentary. This limitation may limit the ability to generalize our findings to CKD patients who do regularly engage in exercise. Further, of those that did complete this maneuver, coherence values remained <0.70, which contrasts with the more robust coherence values (>0.80) observed during the 0.05 Hz maneuver. Despite our reduced sample size and lower coherence observed during this maneuver, group comparisons of the TFA metrics derived from the 0.10 Hz maneuver are similar to those observed during the 0.05 Hz maneuver, supporting the conclusion that cerebral pressure‐flow relationship remains preserved in CKD. Also, we only measured MCAv in one hemisphere and did not perform bilateral monitoring of MCA. Including a combination of right and left MCAv recordings has the potential to confound results due to the cognitive element associated with performing repeated sit‐to‐stand maneuvers. Lastly, although there were no statistically meaningful differences between groups in etCO_2_, it is important to note that the CKD group was relatively hypocapnic compared to the CON group during both repeated sit‐to‐stand maneuvers. These small differences in etCO_2_ may be physiologically relevant, and it is possible that a mild impairment in cerebral autoregulation was obscured in the CKD group due to this relative hypocapnia. Future work should explore this possibility by precisely controlling etCO_2_ during assessment of cerebral autoregulation using end‐tidal forcing to clamp etCO_2_ at a specific target value.

## 5 CONCLUSION

In conclusion, we observed no impairment in dynamic cerebral autoregulation during repeated bouts of sit‐to‐stand in patients with CKD stages III‐IV, when compared to well‐matched controls. These findings suggest that other mechanisms likely contribute to the increased cerebrovascular disease risk exhibited in CKD. Future work should determine if other cerebrovascular regulatory mechanisms such as cerebrovascular CO_2_ reactivity are neurovascular coupling are impaired and related to cerebrovascular disease risk in CKD.

### PERSPECTIVES AND SIGNIFICANCE

Patients with CKD exhibit a substantially elevated risk for cerebrovascular disease. However, cerebral autoregulation is preserved in CKD stages III‐IV suggesting that other mechanisms contribute to this heightened risk. Future work should explore the role of other cerebrovascular regulatory mechanisms in CKD as they relate to stroke risk in this population.

## AUTHOR CONTRIBUTIONS

J.D.S. and J.P. conceived and designed research. J.D.S., T.J. J.J and D.D performed experiments. J.D.S. analyzed data. J.D.S. and J.P. interpreted results of experiments. J.D.S. and J.J. prepared figures. All authors edited and revised manuscript and approved the final version of manuscript.

## FUNDING INFORMATION

This work was supported by the American Heart Association (Career Development Award #929040, PI: J.S.) and the National Institutes of Health Grants (R01HL135183 and R61AT010457 PI: J.P.).
